# BRACAVENIR: an observational study of expectations and coping in young women with high hereditary risk of breast and ovarian cancer

**DOI:** 10.1186/s13053-019-0107-7

**Published:** 2019-02-27

**Authors:** Fabrice Kwiatkowski, Mathilde Gay-Bellile, Pascal Dessenne, Claire Laquet, Véronique Boussion, Marie Béguinot, Marie-Françoise Petit, Anne-Sophie Grémeau, Céline Verlet, Charlotte Chaptal, Marilyn Broult, Sylvie Jouvency, Martine Duclos, Yves-Jean Bignon

**Affiliations:** 1 Oncogenetics department, Comprehensive Cancer Center Jean Perrin, 58, rue Montalembert, 63011 Clermont-Ferrand, France; 2 Senology department, Centre Jean Perrin, Clermont-Ferrand, France; 3 Surgery, Centre Jean Perrin, Clermont-Ferrand, France; 4 Gynecology, CHU Estaing, 1 Place Lucie Aubrac, 63000 Clermont Ferrand, France; 5 Social services, Centre Jean Perrin, Clermont-Ferrand, France; 6 Nutrition, Centre Jean Perrin, Clermont-Ferrand, France; 7 Sports Medicine and functional explorations, CHU Gabriel Montpied, CRNH, INRA, University of Clermont Auvergne, 63000 Clermont-Ferrand, France

**Keywords:** Oncogenetics, Young women, Coping, Hope, Psychodrama, Group sharing, Cancer, Oncology, HBOC

## Abstract

**Background:**

In families with high risk of hereditary breast/ovarian cancer (HBOC), women before age 30 do not yet undergo clinical screening, but they are exposed to contradictory information from diverse sources. They may be presented with surgical prevention options at a key moment of their identity construction, the start of a marital relationship and/or at the onset of procreation projects. We tested an original psychoeducational intervention to help these women better cope with these difficult issues.

**Methods:**

Seven young female counselees (26.4 ± 2.9 years [23–30]) from the Oncogenetics Department at Jean Perrin Comprehensive Cancer Center were enrolled. A weekend group workshop composed of short conferences, group sharing and role playing activities was supervised by a psychotherapist. A longitudinal analysis of questionnaires over one year of follow-up was performed. The Herth Hope Inventory was evaluated, as well as self-esteem, anxiety, perceived control, coping, and quality of life. Participants’ comments were collected by a genetic counselor throughout the workshop.

**Results:**

All participants were BRCA mutation carriers and six had lived with a close relative affected by breast/ovarian cancer. Hope, self-esteem and quality of life increased during the year after the workshop (*p* = 0.0003). Coping by focus on the problem increased in the first 6 months (*p* = 0.011) and returned to baseline values at one year, while coping by focus on emotions decreased steadily (*p* = 0.021). Debriefing from the workshop highlighted the new medical opportunities proposed and the challenges these young women face, such as whether to have prophylactic surgery, and if so before or after having children, and how surgery might affect their relationship with their partner.

**Conclusion:**

A tailored two-day psychoeducational workshop may be sufficient to improve the way young women with BRCA mutations deal with the implications of HBOC risk.

**Trial registration:**

BRACAVENIR was registered in ClinicalTrials.gov with no NCT02705924.

**Electronic supplementary material:**

The online version of this article (10.1186/s13053-019-0107-7) contains supplementary material, which is available to authorized users.

## Background

Young adults carrying mutations favoring the development of cancer encounter various life difficulties, notably questions concerning the future and how to build long-term projects. In western countries, regular screening for the early detection of cancer is the standard of care, but psychosocial consequences remain unaddressed. This is particularly true in hereditary breast ovarian cancer (HBOC) for family members 18–25 years old: women who are not yet screened by mammography or proposed preventive measures such as annexectomy, but who have often already been witness to their mother’s disease and sometimes her death. During this early life period, women are often vulnerable as they face identity issues, the onset of romantic relationships, and questions about future procreation [1–3].

These young women are subject to a plethora of conflicting information and advice spread by the media, websites, internet forums, and family members. The rapid evolution of medicine adds to the confusion, proposing prophylactic surgery, assisted procreation and embryo selection, or promising the advent of gene therapy. Moreover, specialists’ opinions are sometimes discordant. Unfortunately for these young women, such medical measures directly address issues such as self-image, sexuality, and relationships, while these are still in development. A woman’s familial and cultural environment may also influence the complexity of this situation.

We developed a psychoeducational intervention tailored for these young women [4]. A two-day workshop was organized, consisting of short conferences given by experts in each domain, psychological training through role-playing games, and group sharing. To evaluate the impact of this intervention on several cognitive and psychosocial dimensions, self-questionnaires were administered through a dedicated Internet website. Two groups of about 12 participants were initially planned with interventions separated by 6 months (experimental group versus waiting list). Because recruitment was much more difficult than expected, only one group of young counselees was recruited, and the study was analyzed in a cohort design.

## Methods

### Study design

A prospective, mono-center, psychoeducational cohort study was performed. Ethical approval was obtained from the “Comité de Protection des Personnes” in March 2016 (CPP SUD-EST-6: n° IRB00008526) and was registered in ClinicalTrials.gov, no NCT02705924.

### Inclusion criteria

Childless participants 18 to 30 years old belonging to hereditary breast/ovarian cancer (HBOC) families and tested for BRCA mutations (positive or negative), but without personal history of cancer, were recruited. Families negative for BRCA mutations were included if their risk scores were ≥ 6 on the Eisinger scale or ≥ 16 on the Manchester scale [5–7]. To limit transportation costs, participants were recruited from the Auvergne region. Written informed consent was preliminary to any participation.

### Exclusion criteria

Pregnant women were excluded as well as individuals unable to answer questionnaires either for language difficulties or because they could not connect to our website. Psychiatric troubles and/or ongoing related treatments or any treatment incompatible with a 2-day stay in a hydrothermal spa center also prevented entry in the study.

### Objectives

Our primary objective was to observe the evolution of the subjects’ expectations and methods of coping with their cancer risk.

Secondary objectives included [4]:

- Subjects’ improved understanding of genetic and cancer risk information
- Subjects’ improved self-image
- Understanding how these young women respond to issues like marital relationships and family planning, including their emotional and sexual fulfillment
- Higher participation in medical screening programs later in life

### Evaluation criteria

**Fig. 1:**
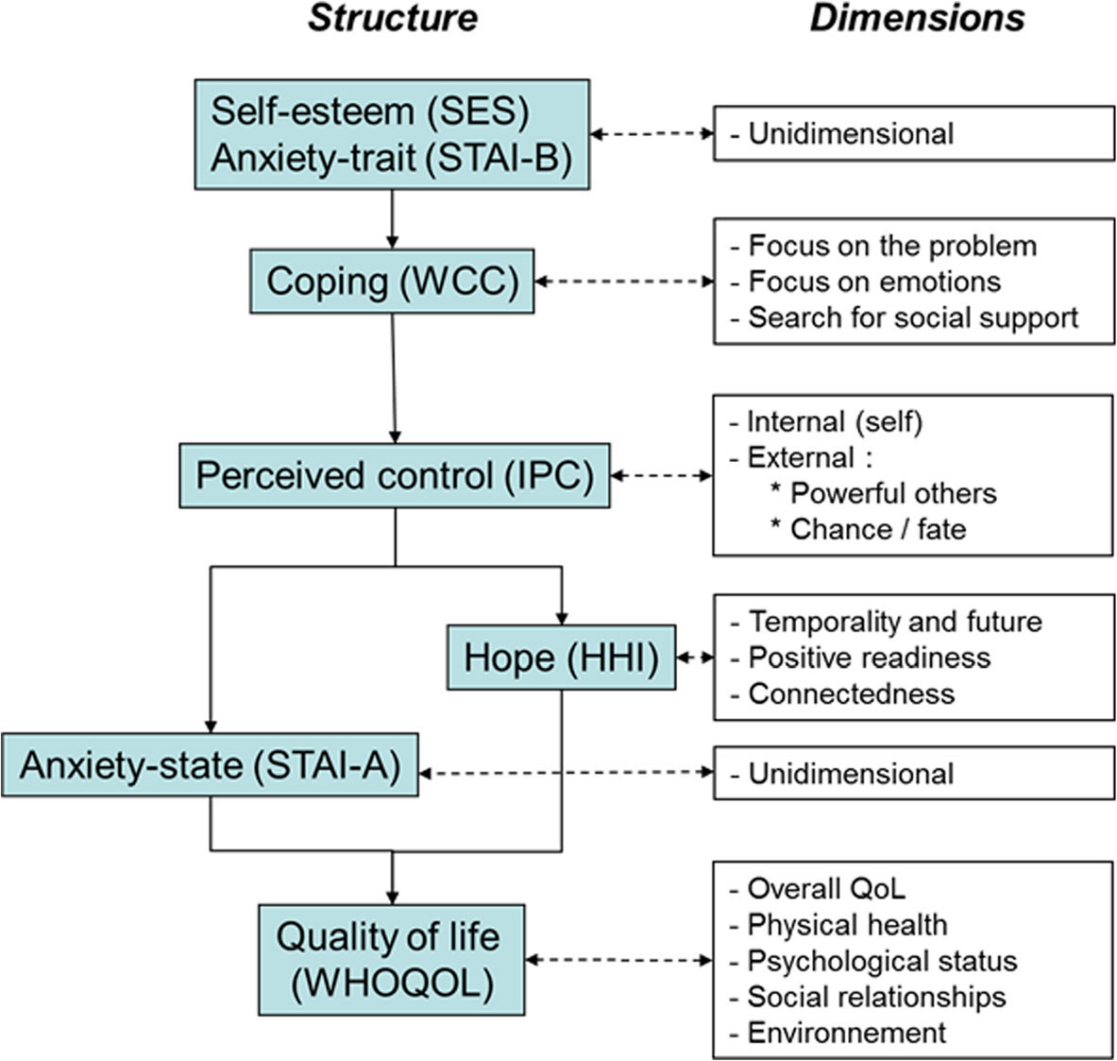
Construct of the psychological model enabling evaluation of the study endpoints

Several self-questionnaires were used to evaluate psychological parameters and their evolution. Responses were collected at four points: at inclusion, at the conclusion of the workshop, then 6 and 12 months after the workshop. A hierarchical model (Fig. 1) was built to represent the main psychosocial dimensions possibly impacted by the information workshop targeting HBOC risk and its management. All questionnaires were translated into French and validated.

We used an array of questionnaires to evaluate personality traits, including Rosenberg’s Self-Esteem Scale [8] and the STAI-B for anxiety [9, 10]. Coping was examined using the WCC questionnaire developed by Folkman and Lazarus [11, 12]. Perceived control, resulting from interactions and feedback between the individual and his environment, was evaluated using Levenson’s IPC [13]. This questionnaire has three dimensions: a locus of control originating in the self, another located outside the individual with two possible sources: “chance/fate” and “powerful others”. We assessed whether HBOC predisposition was considered to belong to the self and whether it reflected the “chance/fate” dimension of IPC. The locus of control may influence two other labile dimensions: anxiety and expectations, particularly in young people. Anxiety was measured using the STAI-A, and expectations were explored using Herth’s Hope Inventory (HHI) [14, 15]. Overall, these psychological dimensions interact with educational and/or socioeconomic levels and the result can be estimated using generalist quality of life (QoL) questionnaires. We chose the World Health Organization questionnaire, WHOQOL [16], which does not focus excessively on health aspects. Global personality questionnaires were not used because we did not believe that a two-day workshop could significantly influence personality.

### Recruitment of participants

**Fig. 2:**
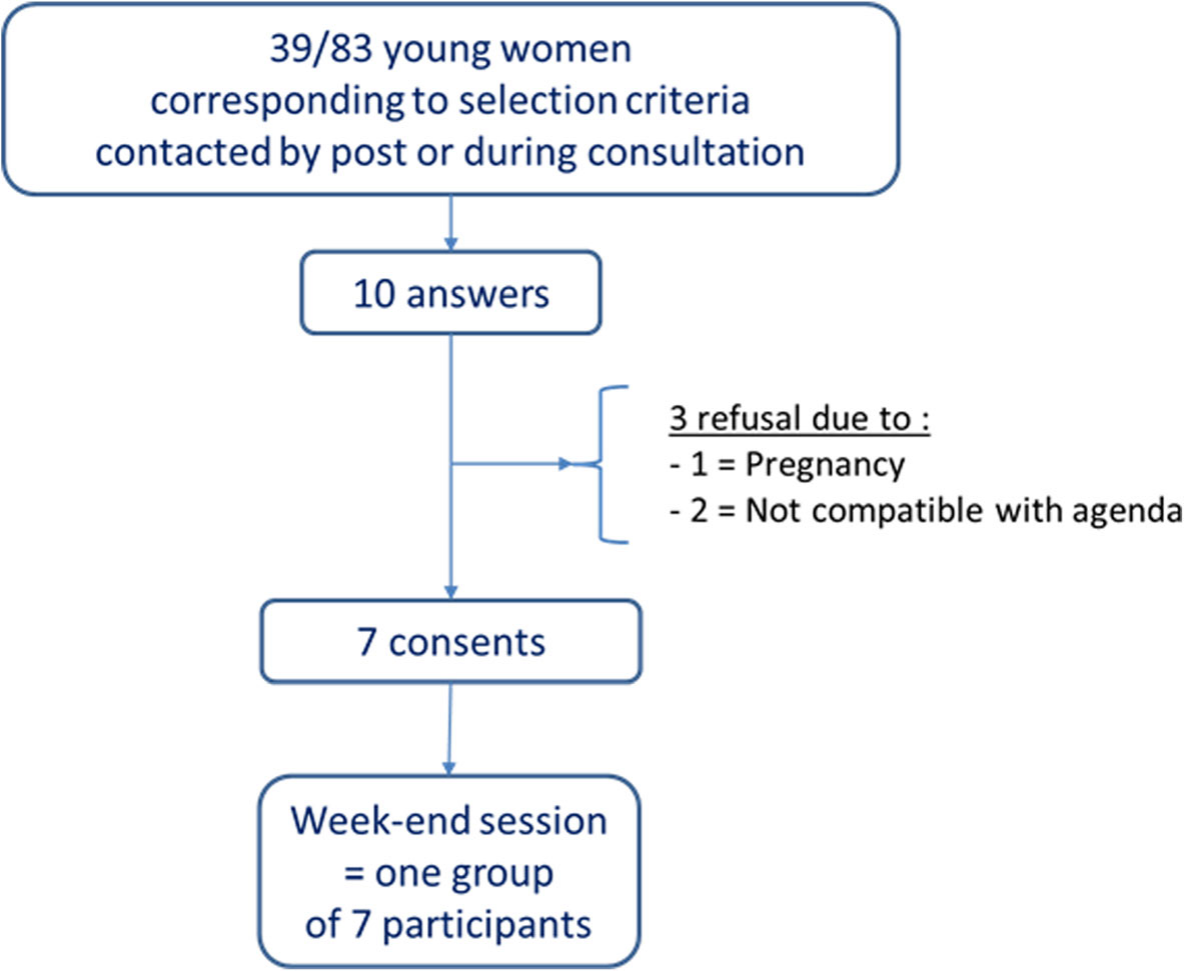
Inclusion flowchart and constitution of the final participants group

Eighty three counselees belonging to HBOC families, aged between 18 and 30 and with a known address, were extracted from the database of the Oncogenetics Department of the Jean Perrin anticancer center. Invitations were sent to the 39 women meeting the inclusion criteria (Fig. 2). Because the workshop was cost-free, at a comfortable spa hotel, we thought that this number would be sufficient to fill two randomized groups of 8 to 12 women. Surprisingly we only could recruit seven participants, despite follow-up mails or phone calls. We therefore established one group with a minimum size to allow group sharing and role games.

Among the reasons explaining the high refusal rate (74% overall) was the feeling that cancer did not concern the candidate at such a young age, and that they had time before acting on it. Others thought that cancer predisposition was not worth spending a weekend listening to experts, or the workshop dates were not compatible with their agenda. Others had children or were pregnant and did not meet the inclusion criteria after all. The age of participants and refusers did not differ (26.4 ± 2.9 versus 27.2 ± 2.9, *p* = 0.46).

### Psychoeducational session

Transportation, meals and lodging at the workshop hotel were paid for by the foundation that funded our project. A documentary about a family with a BRCA mutation was shown (“Life at all costs” by Annick Goutal). There was a 2-h session for Moreno psychodrama with topics chosen by participants, and a group sharing session. Ten conferences lasting 15 to 30 min were given by experts in the following domains:

- Oncogenetics: the mechanics of heredity, mutations in the BRCA and other genes and cancer risk, importance of adapted medical screening and other treatment strategies including prophylactic surgery.
- Epidemiology of HBOC: incidence curves by age for breast and ovarian cancer. Presentation of one of our recent studies exhibiting a fertility advantage in BRCA mutation carriers [17], and of statistics concerning the effect on life expectancy compared to that observed in other diseases and/or deleterious life habits (schizophrenia, obesity, tobacco use …)
- Psychological aspects: review of difficulties faced by members of families with mutations, and in particular the need for psychological support before any prophylactic intervention.
- Surgery: presentation of techniques of breast reconstruction, along with the non-negligible risk of poor outcome. Consequences on breast-feeding, fertility, etc. Review of current French recommendations concerning surgical prophylaxis regarding breast and ovaries, in relation to women’s age and procreation projects.
- Social services: in France, genetic testing, medical screening and prophylactic measures are paid by health insurance. However, indirect costs may be incurred. Aspects regarding loans and confidentiality were recalled.
- Presentation of personalized programs of medical screening, according to cancer risk type (HBOC, HNPCC…)
- Breast health and screening: methods of breast cancer detection (MRI, ultrasound, mammography). Patient’s pathway when a cancer is suspected or diagnosed. Justification of the screening frequency. Special practical session to learn self-palpation of the breast.
- Assisted procreation: possibility of ovarian preservation in case of cancer treated by chemotherapy. Description of how an ovarian sample is obtained after stimulation and how it is re-implanted. France does not allow oocyte donation for BRCA mutation carriers.
- Nutrition: nutritional measures to prevent cancer and also to limit the risk of relapse. Recall of guidelines for better nutrition to control weight and abdominal adiposity. Description of nutrients increasing disease risk and those recommended for better health.
- Physical activity (PA): presentation of the level recommended with proven benefits for prevention and in limiting the risk of relapse. Sedentarity is also a risk factor for many diseases, independently of the level of PA.

### Reporting of participants comments

Throughout the different phases of the workshop, a genetic counselor noted participants’ comments so that a debriefing could be performed retrospectively. Notes were taken during the role playing session, and particularly once the role-play was over, when the group shared their opinions and feelings about what was played, especially regarding echoes in their personal lives. We performed a qualitative analysis of participants’ comments to extract main issues raised.

### Statistical analysis

Quantitative analysis was used for scores obtained by questionnaire.

Categorical variables are described using counts per category and quantitative variables using means ± standard-deviation [range]. Longitudinal analyses of questionnaire scores were tested using one-way ANOVA or Kruskal-Wallis H-test depending on normality and/or homoscedasticity of distributions. Pearson’s correlation test was performed in complement to check the significance of trends over time, when ANOVA or H-test was close to significance. Differences between sub-dimensions of questionnaires were tested using two-way ANOVA. All tests were two-sided and standard *p*-value < 0.05 was used as significance threshold.

Data gathered via the internet website was managed using Ennov Clinical software, version 7.3.1., and statistical calculations were performed with SEM software [18].

## Results

### Participant characteristics

All seven participants were BRCA mutation carriers. Mean age was 26.4 ± 2.9 years, range 23 to 30. Five had a university educational, including two students. Four studied or worked in a medical domain. All had close relatives with a history of cancer, including the mother for five participants, the grandmother for five and an aunt for three. For six participants, more than one close relative had cancer: mainly breast (*N* = 6) or ovarian (*N* = 2). Participants reported other cancer locations in family members, including lung and esophagus. Six participants had at some time shared her daily life with a relative with cancer. The age at cancer diagnosis of the closest relative was 42.0 ± 11.9 [29–65] while participants’ age at the time of these diagnoses was 11.9 ± 7.4 [2–24]. All participants considered their personal cancer risk as either average (*N* = 3) or high (*N* = 4). As a matter of concern, this was rated occasional by four participants, frequent by two others, and permanent for one.

### Analysis of questionnaires

Questionnaires were completed for five participants at all time points (Additional file 1: Table S1). One completed the first two rounds of questionnaires, but was diagnosed with breast cancer shortly before the third round at 6-months post-workshop. Another participant did not answer the 1-year questionnaires.

**Fig. 3:**
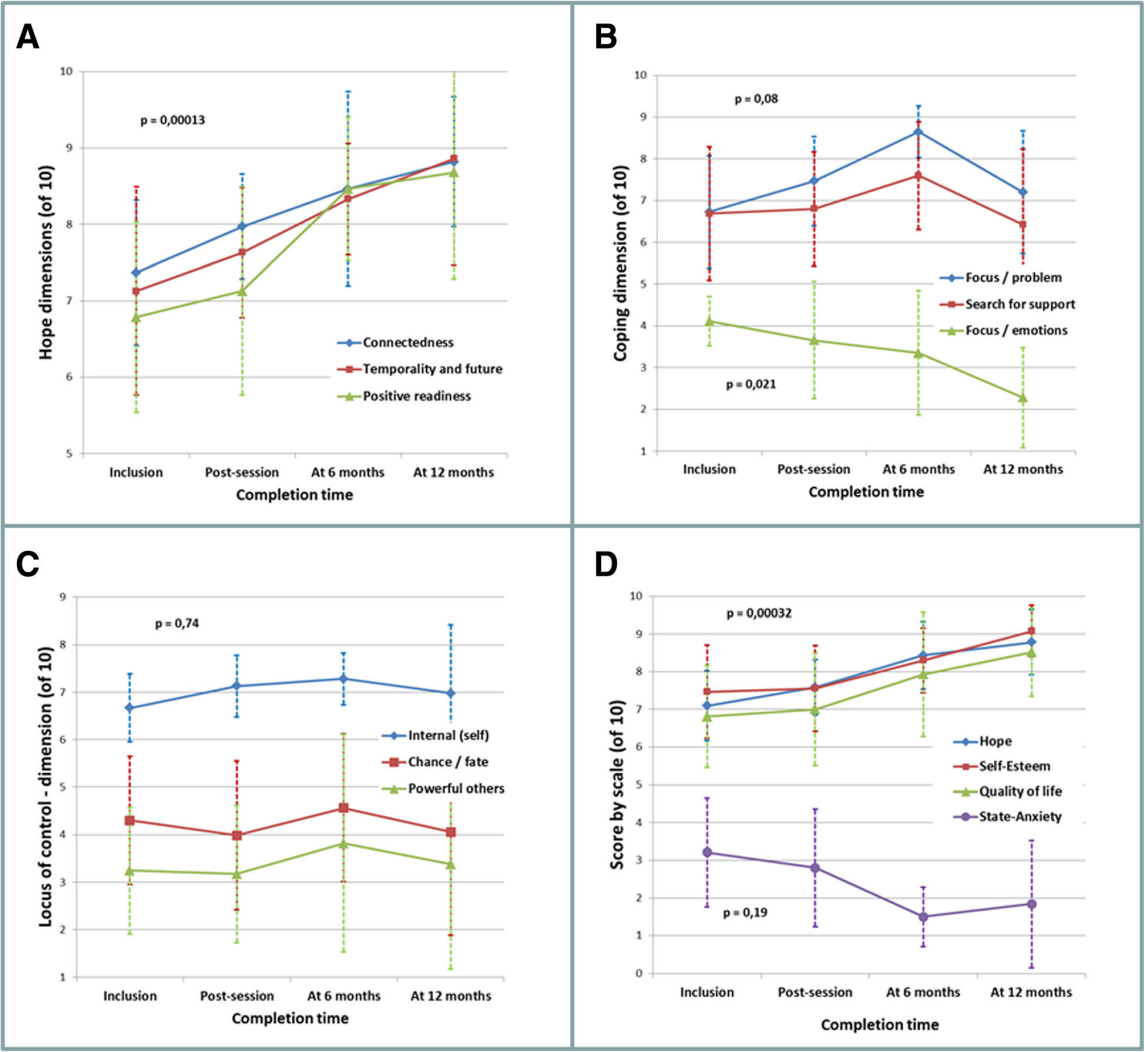
Responses to questionnaires from inclusion to 12-months post-workshop. **a** Herth’s Hope index, **b** Folkman’s Ways of Coping checklist, **c** Levenson’s IPC scale and **d** main global scores. (Error-bars correspond to 95% confidence interval of means and probabilities to time effects)

All three dimensions of Herth’s hope index (HHI) increased over time (Fig. 3 a), confirming the significant global increase of hopefulness following the intervention (*p* = 0.00013); all dimensions increased similarly and no curve-effect was observed (*p* = 0.42). The strongest increase concerned the “positive readiness” component (28%, ANOVA *p* = 0.056; Pearson’s r, *p* = 0.0076). Connectedness and temporality/future increased respectively by 20 and 24% (*p* = 0.01).

Global scores for HHI increased by 24% over the 12-month follow-up period. The associated *p*-value using one-way ANOVA was 0.07 and Pearson’s correlation was 0.02 (blue curve, Fig. 3 d). Self-esteem (22% increase, *p* = 0.10 and 0.015 resp.) and quality of life (25% increase, *p* = 0.24 and 0.04 resp.) followed the same trend. A decrease in state-anxiety by 43% was close to significance (*p* = 0.19 with ANOVA and 0.05 with Pearson’s correlation), and trait-anxiety decreased non-significantly (− 29%, *p* = 0.37 and 0.07 respectively, curve not shown). Altogether, the improvement over 1 year of hope, self-esteem and quality of life was very significant (*p* = 0.00032).

Locus of control was measured by the IPC scale (Fig. 3 c). Internal control scored much higher than “chance/fate” and “powerful others”. This difference was strongly significant (*p* < 10^− 7^) and did not evolve over time (*p* = 0.74).

Coping changed slightly over time (Fig. 3 b). The “focus on the problem” dimension showed a positive trend for 6 months (*p* = 0.011) then returned to baseline values at 1 year; “search for support” exhibited a similar but milder trend. “Focus on emotions” exhibited a steady slope (0.021).

WHOQOL sub-dimensions did not significantly change over time (*p* = 0.54, curves not shown), although the “environment” and “psyche” dimensions exhibited a slight increase over the year. Anxiety levels at inclusion evaluated with the trait-STAI questionnaire were low: five participants had very low or low anxiety, while two had medium anxiety levels.

### Reporting of participants’ comments

Participants’ comments concerned several topics summarized below:

- Genetics:
  - ○ Some regretted having genetic testing so soon.
  - ○ All participants stated that the announcement of their genetic status made them cry, either immediately or shortly after. This announcement was experienced as a real, and often brutal, psychological trauma.
  - ○ Fundamental genetics information was requested, notably What about the hereditary risk transmitted by males? Does everyone have a BRCA gene? Are *de-novo* mutations possible?
- Familial cancer experience: all reported that they had experienced the death of a close relative because of breast or ovarian cancer. Three lost their mother before their teenage years. For the others, the idea of developing cancer themselves would be similar to making their mother re-live her own illness. Prophylactic surgery appeared to be the only way to avoid such a situation.
- Prophylactic breast surgery:
  - ○ Mastectomy with skin or nipple areolar plate preservation, as presented during the surgeon’s conference, was a discovery for the participants. This was a matter of intense debate, in particular regarding the residual risk when the whole breast is not removed, and whether preserving body image is a priority over maximal cancer risk reduction.
  - ○ Many expressed their need for real examples of prophylactic breast surgery with reconstruction. One declared: “I shall volunteer to show my result to women who ask me”.
  - ○ Some felt guilty for their uncertainty about the “right” decision to make, for being unable to totally master their life, for not being sure of themselves.
- Spouse/Boyfriend:
  - ○ Preparing a partner for the news was a significant task, especially for single women. One woman raised by her widower father explained her partner’s fear of remaining alone as a widower and managing their children. The partner observed his father-in-law’s model, which he admired without being willing to take on a similar hardship. He did not feel he had the courage to take care of children alone.
  - ○ “I will try to let him consider the risk for the future: evoking the BRCA risk with a new boyfriend is difficult, but it is a proof of trust.”
  - ○ “If my boyfriend does not agree with my decision for surgery, let him leave!”
  - ○ There was much discussion of how prophylactic surgery would change the couple’s relationship. “If this induces a separation, it’s because there was no love before; the surgery is just a trigger.” “As a matter of fact, surgery is a weakening element. I’m afraid of what happens after: this questions the solidity of the couple.” “Asking for prophylactic surgery is like heading into the unknown. If my partner does not adhere, I will hesitate even more.” “How will we cope with the change? My boyfriend said to me: I will have to say goodbye to your nipples.” “To see a breast without a nipple is difficult for a woman: what must it be for a man?”
- Childbearing:
  - ○ Participants requested information as to whether pregnancy was a risk factor for cancer, and whether medical screening changed during pregnancy.
  - ○ Some found it difficult to decide whether to have children before or after prophylactic surgery. In particular, prophylactic mastectomy precludes any future breast-feeding, which has a protective effect against cancer risk.
  - ○ Participants were cognizant of the opportunities presented by modern medical care (screening, prophylactic surgery…) unavailable to their mothers.
  - ○ No one spoke of the utility of prenatal or preimplantation diagnosis. Although a significant burden, BRCA mutations were not considered stigmatizing or as an injustice.

## Discussion

Considering our recruitment difficulties, young women in HBOC families may not be ready to spend time “bothering” about their cancer risk at this period of their lives,. At their young age, their low cancer risk was likely not a priority issue. This contrasted to the proactive attitude of our participants, many of whom worked in a medical field and/or were highly educated. This attitude was not associated with an anxious background: only two participants had a medium trait-anxiety level, while the five others had either low or very low trait-anxiety levels. In a study by Listøl et al. [19] that tested the impact of a standardized educational course on BRCA-mutated patients, it was noted that this kind of intervention selects highly educated participants. In their study of 100 women (mean age = 46, range = [26–69]), 50% had a university degree. In contrast with our participants, their level of anxiety was quite high and in particular “significantly higher than reported in earlier studies of individuals seeking genetic counseling for hereditary cancer”.

Another characteristic of the participants made the group unrepresentative of young women at risk: although the study was open to women in families without BRCA mutations, only women positive for a mutation participated. This selection bias is not surprising and we showed in another survey that members belonging to HBOC families where no mutation was found tend to seek less information about their cancer risk than mutation carriers [20]. It is likely that the uncertainty about the genetic diagnosis has an impact on a cognitive level, preventing these patients from reacting to their familial risk more objectively and proactively.

Our participants were in search for a “solution” to their issue. Gathering all possible information was one way of managing their cancer risk. However, with more information they then faced further difficult choices: whether to have a prophylactic intervention, and if so, before or after childbearing. Moreover, the evolution of surgical practices has brought new controversies, such as skin or nipple areolar plate preservation (SNAPP). Certainly, the role of the surgeon is essential in making this decision, and the free discussion with the young female surgeon during the session was very important from the participants’ point of view. Two participants decided on surgery without SNAPP a few months after the session: For them, there should be no compromise with cancer risk, and the fact that one of the other participants was diagnosed with breast cancer soon after the session seemed to credit their choice.

In deciding on prophylactic mastectomy, the participants considered issues beyond the obvious benefit to themselves in reducing their cancer risk versus the medical risks of the intervention. The inability to breast-feed future babies seemed to be of secondary importance, while the attitude of the partner had both positive and negative aspects. Prophylactic mastectomy with reconstruction alters a woman’s body image and femininity, both for herself and for her partner. On the other hand, it reduces the perspective for the partner to see their wife die prematurely. It also prevents the participants’ mother, if alive, to experience a second cancer via her daughter. Aside from these considerations, other doubts surround the consequences of the surgery, which may induce changes that cannot be anticipated and that threaten the relationship: in all cases, this constitutes a real test for reciprocal feelings and confidence. All the participants downplayed any anticipated negative effects for themselves, focusing instead on their partner.

The session dedicated to role playing and group sharing enabled participants to express their feelings about the consultation providing genetic testing. All the participants reported receiving their molecular diagnosis as BRCA mutation carriers as traumatic, even “brutally” so. Although geneticists are aware of such issues, participants’ declarations tended to show that the distress is generally much deeper than expected. The duration of this trauma has been discussed, some authors considering it to be transient [21, 22] while others report high level of distress, anxiety, depression and/or anger still 1 month after announcement [23, 24], in particular in young counselees from mutated families coming for a targeted test. This was the case of our participants. Although this psychological impact seems to disappear in less than 1 year [22], we think that some measures should be taken in order to diminish the distress generated by the diagnosis announcement.

The results drawn from the questionnaires were unexpected considering the small sample size. Overall, the psychoeducational session proved to be efficient for hopefulness (HHI), coping (WCC) and overall quality of life (WHOQOL). In particular, participants seemed to be more positive regarding their future, and exhibited a better capacity to face their cancer risk (decrease of the focus on emotions).

The overall result of the week-end session was positive and confirmed our goal: bringing to these young women a wide spectrum of information and helping them to express their distress seemed to change their attitudes in a significant and positive manner. This suggests that we should not change the content of our intervention but perhaps recruit slightly older women (up to 40 years) who have new childbearing projects and/or question the cancer risk they transmit to their children.

Aside from the small sample size and thus the limited confidence that can be given to results, the main weakness of our study seems to be the lack of representativeness of our young BRCA carriers. The results might have been different for young BRCA mutation carriers declining participation. Our participants were already invested in the resolution of their genetic problem. Their high educational level was corroborated by their locus of control which was mainly internal and their coping style not very emotional. In concordance with Listøl et al. [19], our study suggests that such workshops may be suited for a limited population of highly educated women seeking information.

## Conclusion

Young females exposed to HBOC, partly due to lethal familial events that disrupted their childhood, see their psyche strongly modulated. Our results suggest that a weekend workshop dedicated to high quality information and favoring individual expression can significantly improve what they know about their cancer susceptibility, they feel and how they live it, and likely it can help them figure out the most adapted solution enabling them to handle their cancer risk. An extension of this study is planned, targeting HBOC women up to age 40 who already have children and/or with a new child project, and who have questions on heredity issues and/or possible present or future prevention measures for themselves and their children.

## Additional file

Additional file 1: **Table S1.** Detail of scores by questionnaire and by completion time. (DOCX 21 kb)

## Abbreviations

ANOVA: Analysis of variance
HBOC: Hereditary breast/ovarian cancer
HHI: Hert’s Hope Index
HNPCC: Hereditary non polyposis colorectal cancer
IPC: Internal, Powerful others, Chance (Levenson’s IPC scale)
PA: Physical activity
QoL: Quality of life
STAI: Strait-trait anxiety inventory
WCC: Ways of Coping Checklist
WHOQOL: World Health Organization Quality of Life
