# Supplementary material for: BRACAVENIR: an observational study of expectations and coping in young women with high hereditary risk of breast and ovarian cancer
Source: Hered Cancer Clin Pract. 2019 Feb 27;17:7. doi: 10.1186/s13053-019-0107-7 (PMC6391839; doi:10.1186/s13053-019-0107-7)
Supplement: Supplementary file 1 — Table S1. Detail of scores by questionnaire and by completion time. (DOCX 21 kb) [file 13053_2019_107_MOESM1_ESM.docx]

**Appendix**

Table 1: detail of scores by questionnaire and by completion time.

| **Questionnaires** | **Baseline** | | | **Post-workshop** | | | **6-months** | | | **12 months** | | | **Trend** | |
| --- | --- | --- | --- | --- | --- | --- | --- | --- | --- | --- | --- | --- | --- | --- |
|  | mean | SD | 95%-CI | mean | SD | 95%-CI | mean | SD | 95%-CI | mean | SD | 95%-CI | p-value | variation |
| **HHI - Herth's Hope Index** | **7.10** | 1.00 | [6.18-8.02] | **7.59** | 0.79 | [6.86-8.32] | **8.43** | 0.86 | [7.53-9.33] | **8.78** | 0.69 | [7.93-9.63] | 0.0015 | 23.7% |
| connectedness | **7.37** | 1.03 | [6.42-8.32] | **7.97** | 0.75 | [7.28-8.66] | **8.47** | 1.21 | [7.2-9.74] | **8.82** | 0.69 | [7.97-9.67] | 0.012 | 19.7% |
| temporality and future | **7.13** | 1.48 | [5.77-8.49] | **7.63** | 0.92 | [6.78-8.48] | **8.33** | 0.69 | [7.6-9.06] | **8.86** | 1.12 | [7.46-10.26] | 0.01 | 24.3% |
| Positive readiness | **6.79** | 1.35 | [5.54-8.04] | **7.13** | 1.48 | [5.77-8.49] | **8.47** | 0.90 | [7.53-9.41] | **8.68** | 1.13 | [7.28-10.08] | 0.008 | 27.8% |
|  |  |  |  |  |  |  |  |  |  |  |  |  |  |  |
| **SES - Self Esteem** | **7.47** | 1.34 | [6.23-8.71] | **7.56** | 1.24 | [6.42-8.7] | **8.30** | 0.82 | [7.44-9.16] | **9.08** | 0.55 | [8.39-9.77] | 0.015 | 21.6% |
|  |  |  |  |  |  |  |  |  |  |  |  |  |  |  |
| **STAI - Anxiety** State | **3.21** | 1.57 | [1.76-4.66] | **2.80** | 1.69 | [1.23-4.37] | **1.50** | 0.75 | [0.71-2.29] | **1.84** | 1.36 | [0.15-3.53] | 0.05 | -42.7% |
| Trait | **3.67** | 1.25 | [2.53-4.84] | **3.43** | 0.88 | [2.62-4.24] | **2.83** | 1.18 | [1.60-4.08] | **2.62** | 0.87 | [1.54-3.70] | 0.07 | -28.6% |
|  |  |  |  |  |  |  |  |  |  |  |  |  |  |  |
| **WHOQOL - quality of life** | **6.81** | 1.46 | [5.46-8.16] | **7.00** | 1.61 | [5.51-8.49] | **7.93** | 1.57 | [6.28-9.58] | **8.52** | 0.94 | [7.35-9.69] | 0.04 | 25.1% |
| **WCC - ways of coping** |  |  |  |  |  |  |  |  |  |  |  |  |  |  |
| Focus on the problem | **6.73** | 1.46 | [5.38-8.08] | **7.47** | 1.16 | [6.4-8.542] | **8.65** | 0.59 | [8.03-9.27] | **7.20** | 1.18 | [5.73-8.67] | 0.22 | 7.0% |
| Search for social support | **6.69** | 1.73 | [5.09-8.29] | **6.80** | 1.48 | [5.43-8.17] | **7.60** | 1.23 | [6.31-8.89] | **6.42** | 1.47 | [4.6-8.24] | 0.65 | -4.0% |
| Focus on emotions | **4.11** | 0.64 | [3.51-4.71] | **3.66** | 1.51 | [2.26-5.06] | **3.35** | 1.42 | [1.86-4.84] | **2.28** | 0.96 | [1.09-3.47] | 0.02 | -44.5% |
| **Locus of control** |  |  |  |  |  |  |  |  |  |  |  |  |  |  |
| Internal | **6.67** | 0.78 | [5.95-7.39] | **7.13** | 0.70 | [6.48-7.78] | **7.28** | 0.52 | [6.73-7.83] | **6.98** | 1.16 | [5.54-8.42] | 0.44 | 4.6% |
| Powerful others | **4.30** | 1.45 | [2.95-5.65] | **3.99** | 1.69 | [2.42-5.56] | **4.57** | 1.49 | [3.01-6.13] | **4.06** | 1.76 | [1.88-6.24] | 0.73 | -5.6% |
| Chance / Fate | **3.24** | 1.44 | [1.9-4.58] | **3.17** | 1.56 | [1.73-4.61] | **3.82** | 2.18 | [1.53-6.11] | **3.38** | 1.78 | [1.18-5.58] | 0.98 | 4.3% |

* SD = standard deviation; 95%-CI = 95% confidence interval of means. P-value = probability associated to a positive or negative trend of outcomes.
 Percent variations (column trend) correspond to differences between 1-year and baseline values
